# The Role of Sensory Nerves in Dental Pulp Homeostasis: Histological Changes and Cellular Consequences after Sensory Denervation

**DOI:** 10.3390/ijms25021126

**Published:** 2024-01-17

**Authors:** Chunmeng Wang, Xiaochen Liu, Jiani Zhou, Qi Zhang

**Affiliations:** Department of Endodontics, Stomatological Hospital and Dental School of Tongji University, Shanghai Engineering Research Center of Tooth Restoration and Regeneration, No.399 Yanchang Middle Road, Jing’an District, Shanghai 200072, China

**Keywords:** sensory nerves, homeostasis maintenance, pulp degeneration, dental pulp cells, cellular senescence

## Abstract

Homeostatic maintenance is essential for pulp function. Disrupting pulp homeostasis may lead to pulp degeneration, such as fibrosis and calcifications. Sensory nerves constitute a crucial component of the dental pulp. However, the precise involvement of sensory nerves in pulp homeostasis remains uncertain. In this study, we observed the short-term and long-term histological changes in the dental pulp after inferior alveolar nerve transection. Additionally, we cultured primary dental pulp cells (DPCs) from the innervated and denervated groups and compared indicators of cellular senescence and cellular function. The results revealed that pulp fibrosis occurred at 2 w after the operation. Furthermore, the pulp area, as well as the height and width of the pulp cavity, showed accelerated reductions after sensory denervation. Notably, the pulp area at 16 w after the operation was comparable to that of 56 w old rats. Sensory denervation induced excessive extracellular matrix (ECM) deposition and increased predisposition to mineralization. Furthermore, sensory denervation promoted the senescence of DPCs. Denervated DPCs exhibited decelerated cell proliferation, arrest in the G2/M phase of the cell cycle, imbalance in the synthesis and degradation of ECM, and enhanced mineralization. These findings indicate that sensory nerves play an essential role in pulp homeostasis maintenance and dental pulp cell fate decisions, which may provide novel insights into the prevention of pulp degeneration.

## 1. Introduction

The dental pulp is a specialized loose connective tissue containing nerve fibers, blood vessels, and a significant number of cells [1]. The concerted effort of multiple components maintains pulp homeostasis and guarantees the proper functioning of the pulp [2]. The dental pulp is highly innervated, with nerves constituting approximately 40% of the volume of the pulp chamber, predominantly comprising sensory nerve fibers originating from the trigeminal ganglia [3,4]. A range of studies have shown that sensory nerves play a crucial role in maintaining the homeostasis of various tissues, and sensory denervation results in degenerative changes [5,6,7,8]. For example, knocking out TrkA^+^ sensory nerve fibers disrupts bone homeostasis, which exhibits diminished bone formation ability and the early onset of osteoporosis [9]. The absence of sensory innervation in skeletal muscle leads to a reduction in muscle size and performance, resulting in significant muscle atrophy [10]. Sensory denervation in the disc leads to accelerated and more severe disc stenosis and disrupted matrix homeostasis [11]. Similarly, an imbalance in pulp homeostasis can result in pulp degeneration, including fibrosis and calcification [12,13]. Despite various studies indicating a notable decline in nerve fiber density in aging pulp, accompanied by a high occurrence of pulp calcification [14,15,16], there remains inadequate evidence regarding the involvement of sensory nerves in pulp homeostasis. Therefore, the role of sensory nerves in pulp homeostasis and the relationship between sensory denervation and pulpal degeneration deserve further investigation.

Dental pulp cells (DPCs), the predominant cell lineage within dental pulp, are essential for maintaining the equilibrium of the extracellular matrix (ECM) [17,18,19]. When these cells undergo aberrant changes, an excessive accumulation of ECM and an elevation in mineralized deposits occur, resulting in the onset of pulp degeneration [20,21]. The impact of abnormal sensory innervation on tissue-specific cells may lead to the disruption of the ECM balance in degenerative diseases. Spinal stenosis is accelerated in intervertebral disc degeneration due to reduced chondroitin sulfate synthesis by sensory-denervated nucleus pulposus cells [11]. Aside from this, sensory hyperinnervation coincides with the deposition of type I collagen expressed by synoviocytes, thereby promoting the development of synovial fibrosis in osteoarthritis [22]. In the dental pulp, a complex network of sensory nerves extensively innervates DPCs, forming an integral component of their extracellular microenvironment [4]. However, the precise impact of sensory nerves on the fate of DPCs remains inadequately elucidated. Accordingly, whether sensory nerves maintain pulp homeostasis by influencing DPCs also warrants being examined in depth.

Therefore, we constructed a model of pulpal sensory denervation by inferior alveolar nerve dissection and conducted short-term and long-term observations. In vitro, we performed a primary DPC culture from innervated and denervated groups and conducted the associated analysis. We demonstrate that sensory nerves play an essential role in pulp homeostasis maintenance.

## 2. Results

### 2.1. Sensory Denervation Accelerates Pulp Obliteration

**Figure 1:**
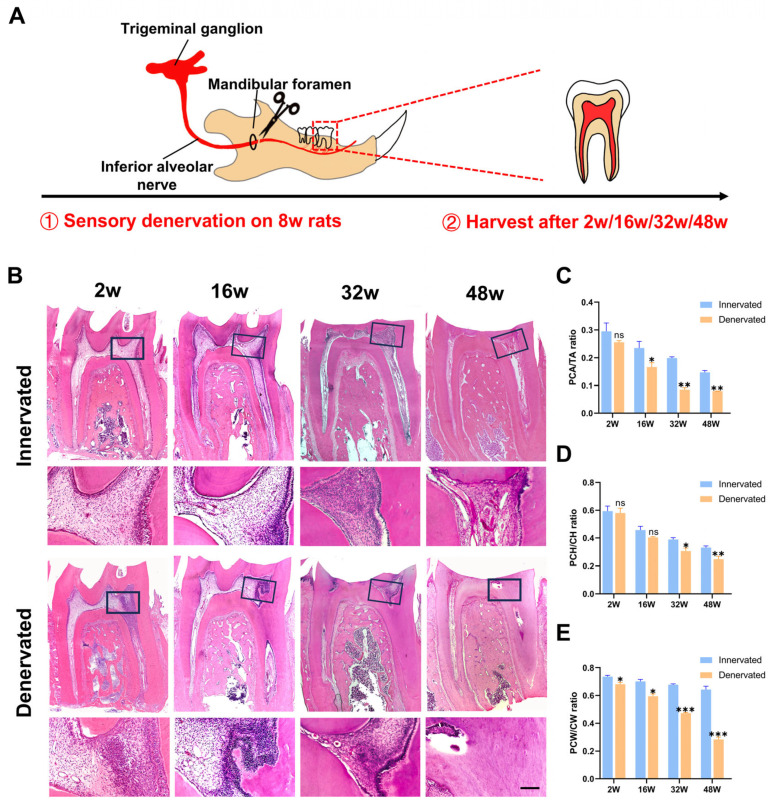
Sensory denervation accelerates pulp obliteration. (**A**) Schematic illustrating the denervation strategy (n = 4). (**B**) HE staining of mandibular first molars of innervated and denervated groups at 10 w, 24 w, 40 w, and 56 w. Boxed regions are magnified; scale bar = 100 µm. (**C**) Pulp chamber area/tooth area (PCA/TA) ratio of the teeth (ns, no significant difference; * *p* < 0.05 and ** *p* < 0.01). (**D**) Pulp chamber height/crown height (PCH/CH) ratio of the teeth (ns, no significant difference; * *p* < 0.05 and ** *p* < 0.01). (**E**) Pulp chamber width/crown width (PCW/CW) ratio of the teeth (* *p* < 0.05 and *** *p* < 0.001).

We constructed a model of inferior alveolar nerve transection to disrupt the sensory innervation of mandibular molars in 8-week-old rats and collected samples at 2, 16, 32, and 48 weeks after the operation, when the rats were 10, 24, 40, and 56 weeks old, respectively (Figure 1A). The unoperated rats were set as the control group with normal innervation. We observed a progressively smaller pulp area and narrower pulp chamber in the normal mandibular first molars with age (Figure 1B–D). It was shown that sensory denervation accelerated the degenerative changes in the dental pulp, and we found that 2 w after denervation, pulp fibrosis occurred in some of the molars; 16 w after denervation, the pulp showed extensive root canal calcification, and the pulp areas are similar to the innervated molars of old rats at 56 w. Further, 32 w and 48 w after denervation, the calcified area increased gradually. The narrowing of the pulp chamber in the innervated group was mainly due to the loss of height, while the height and width of the pulp chamber were reduced in the denervated group. The denervated pulp showed more prominent fibrosis and calcification compared with the innervated pulp of all ages (Figure 1B–D).

The aforementioned results provide evidence of a strong correlation between sensory nerves and degenerative alterations in dental pulp. Having established that dental pulp changes can occur within a mere 2 weeks following surgery, we consistently employed mandibular first molars at this time point for all subsequent investigations into the specific details underlying pulp degeneration induced by sensory denervation.

### 2.2. Sensory Denervation Induces Premature Pulp Aging and Pulp Degeneration

**Figure 2:**
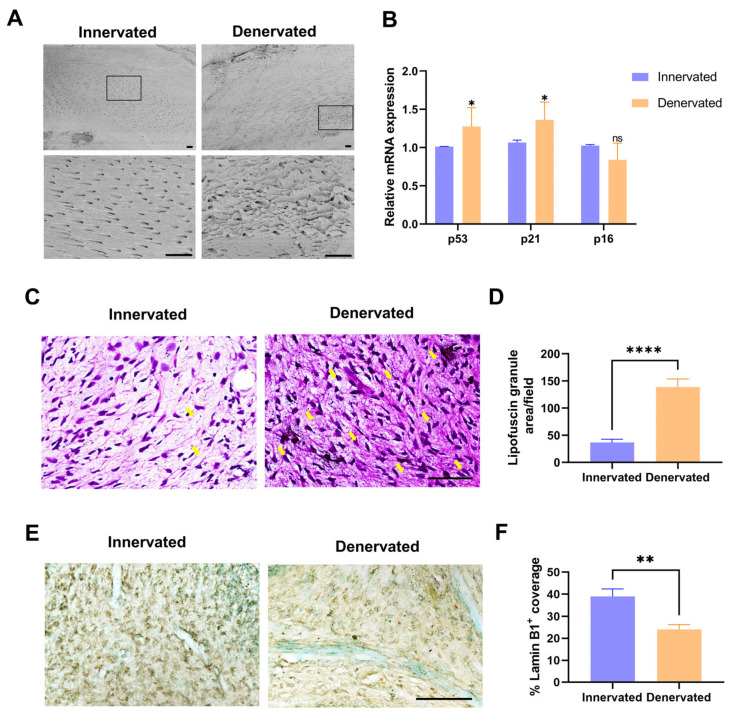
Sensory denervation increases the levels of senescence markers in the dental pulp. (**A**) Scanning electron microscopy of dentin in the innervated and denervated groups. Scale bar = 10 µm. (**B**) RT-qPCR analysis of p53, p21, and p16 mRNA expression of pulp tissue (ns, no significant difference; * *p* < 0.05, n = 3). (**C**) Lipofuscin staining of innervated and denervated groups. Yellow arrows show clusters of lipofuscin granules. Scale bar = 100 µm. (**D**) Quantification of the staining in (**C**) (**** *p* < 0.0001, n = 3). (**E**) Immunohistochemical staining of Lamin B1 in the innervated and denervated groups. Scale bar = 100 µm. (**F**) Quantification of the staining in (**E**) (** *p* < 0.01, n = 3).

We used a scanning electron microscope (SEM) to observe the morphology of the dentin surface (close to the pulp chamber) of the innervated and denervated teeth. The surface of the denervated dentin appeared partially rough and uneven, but there was no significant change in the diameter of the dentin tubules, nor was there any apparent obstruction (Figure 2A).

The total RNA was extracted from the dental pulp tissues from both groups, cDNA was prepared, and the levels of senescence-related genes p53, p21, and p16 were assessed by qPCR [23]. There was markedly increased p53 and p21 gene expression, and no differences were observed in the p16 levels (Figure 2B). We further performed the Ziehl–Neelsen staining (lipofuscin staining) on the pulp slides, indicating senescence of the denervated pulp, which showed the massive accumulation of lipofuscin (Figure 2C,D). The immunohistochemical staining revealed that Lamin B1 expression was decreased in the denervated pulp, which also demonstrated that sensory denervation promotes premature pulp aging (Figure 2E,F) [24].

**Figure 3:**
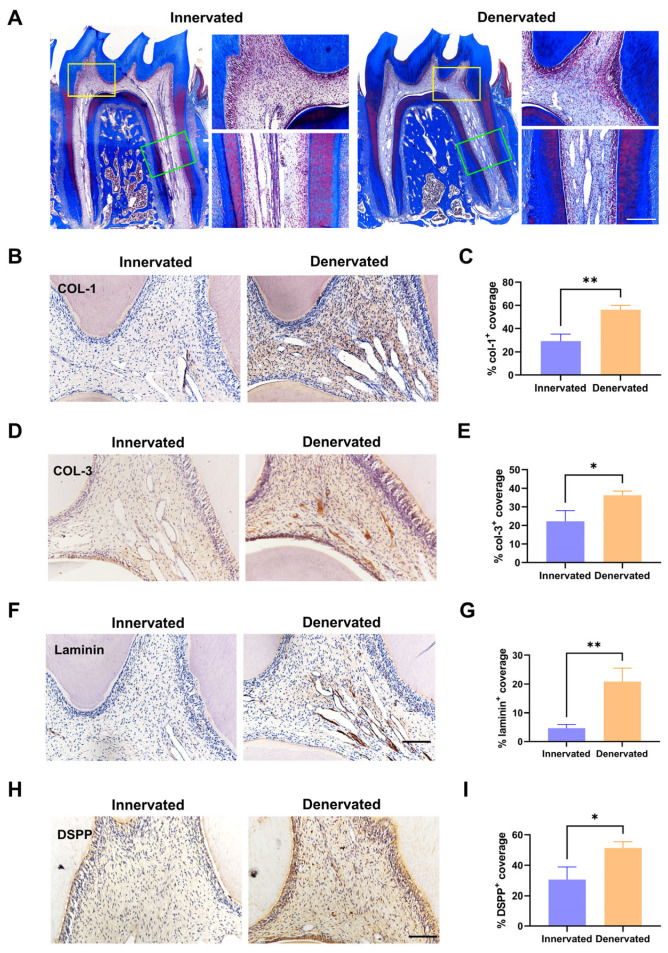
Sensory denervation induces excessive deposition of extracellular matrix and increased predisposition to mineralization. (**A**) Masson staining in the innervated and denervated groups. The yellow boxes indicate the pulp chamber of magnification. The green boxes indicate the root canal of magnification. Scale bar = 100 µm. (**B**) Immunohistochemical staining of COL-1 in the innervated and denervated groups. Scale bar = 100 µm. (**C**) Quantification of the staining in (**B**) (** *p* < 0.01, n = 3). (**D**) Immunohistochemical staining of COL-3 in the innervated and denervated groups. (**E**) Quantification of the staining in (**D**) (* *p* < 0.05, n = 3). (**F**) Immunohistochemical staining of laminin in the innervated and denervated groups. (**G**) Quantification of the staining in (**F**) (** *p* < 0.01, n = 3). (**H**) Immunohistochemical staining of DSPP in the innervated and denervated groups. (**I**) Quantification of the staining in (**H**) (* *p* < 0.05, n = 3).

One of the characteristic features of pulp degeneration is collagen deposition. We performed Masson’s trichrome staining to evaluate the extent of collagen deposition/fibrosis, which revealed a large amount of blue-stained collagen fibers in the pulp chamber and root canal of the denervated tooth (Figure 3A). In order to assess the alteration of the ECM components of the denervated group, the expression of COL-1, COL-3, and laminin was then identified by immunohistochemistry staining; there were significantly increased expressions of all the above (Figure 3B–G). In addition, we also detected the increased expression of the vital protein DSPP, which is involved in the process of odontogenic differentiation, indicating an increased predisposition to mineralization after sensory denervation (Figure 3H,I).

### 2.3. Sensory Denervation Promotes the Senescence of Dental Pulp Cells

**Figure 4:**
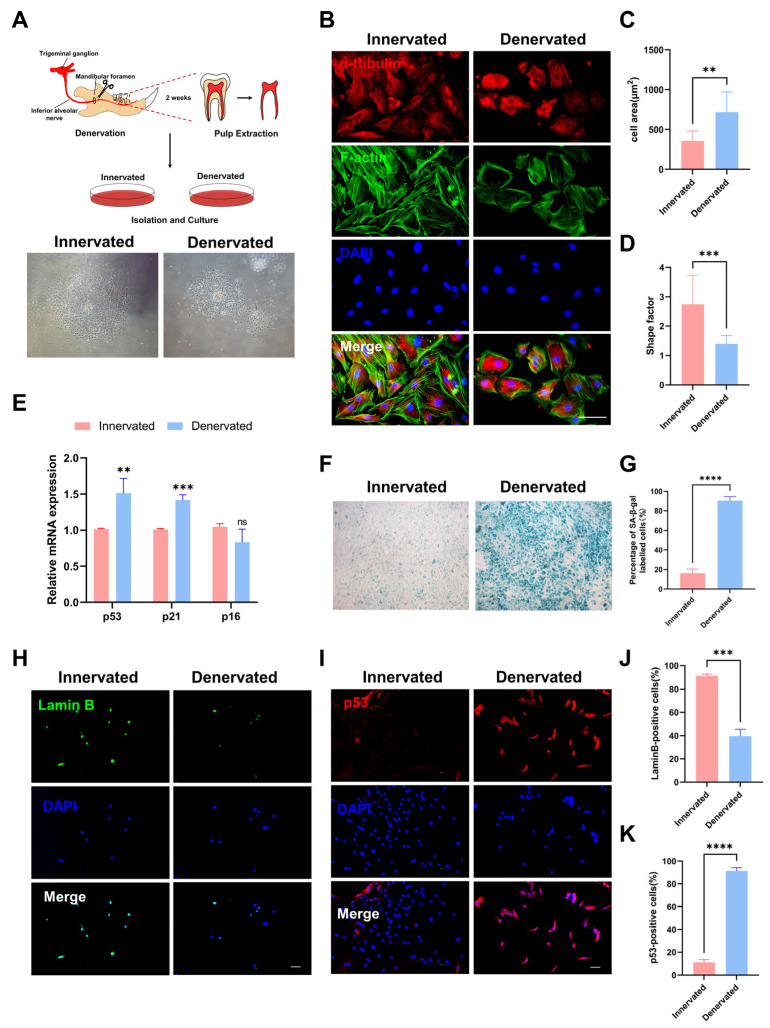
Sensory denervation promotes the senescence of dental pulp cells. (**A**) Primary culture of dental pulp cells (DPCs) from rat mandibular first molar. (**B**) Cytoskeletal changes were assessed by immunofluorescence staining for F-actin (phalloidin, green) and α-tubulin (red). Scale bar = 100 µm. (**C**) Cell area is the area of a cross-section of the cell soma (** *p* < 0.01, n =15). (**D**) The shape factor is the ratio of the major to the minor axis (*** *p* < 0.001, n = 15). (**E**) RT-qPCR analysis of p53, p21, and p16 mRNA expression of DPCs. (**F**) Senescence-associated β-galactosidase (SABG) staining. (**G**) Quantification of the staining in (**F**) (**** *p* < 0.0001, n = 3). (**H**) Immunofluorescence staining of Lamin B1 (green) in the DPCs of innervated and denervated groups. Scale bar = 100 µm. (**I**) Immunofluorescence staining of p53 (red) in the DPCs of innervated and denervated groups. Scale bar = 100 µm. (**J**) Quantification of the staining in (**H**) (*** *p* < 0.001, n = 3). (**K**) Quantification of the staining in (**I**) (**** *p* < 0.0001, n = 3).

DPCs play a crucial role in maintaining the equilibrium of the ECM [17]. To explore the alteration of cellular activity, the DPCs were isolated from the first mandibular molars of the rats in the innervated and denervated groups. The speed of the denervated DPCs that climbed out from the tissue blocks was slower than the normal DPCs (Figure 4A).

We assessed the 2D characteristics and cell appearance via immunofluorescence staining for F-actin and α-tubulin firstly. The statistical analysis indicated that the denervated group tended to have an enlarged occupied area and a decreased cell aspect ratio in comparison to the innervated group (Figure 4B–D). Senescence causes profound changes in cell morphology, such as a flattened, irregular shape or enlarged cell size [24,25]. Next, the qPCR examination showed that the levels of the senescence-related genes p53 and p21 were markedly increased and no differences were observed in the p16 levels, which was consistent with that observed in vivo (Figure 4E). The results of the β-galactosidase staining indicated a significant increase in the proportion of β-galactosidase-positive cells stained in blue in the denervated DPCs (Figure 4F,G). Lower Lamin B1 leads to cellular senescence and p53 is a critical regulator of senescence [26,27]. Therefore, we performed immunofluorescence staining of Lamin B1 and p53 for both groups. The DPCs of the denervated group exhibited low expression of Lamin B1 and high expression of p53 (Figure 4H–K).

### 2.4. Denervated DPCs Show Cell Cycle Arrest and Imbalance in Synthesis and Secretion

**Figure 5:**
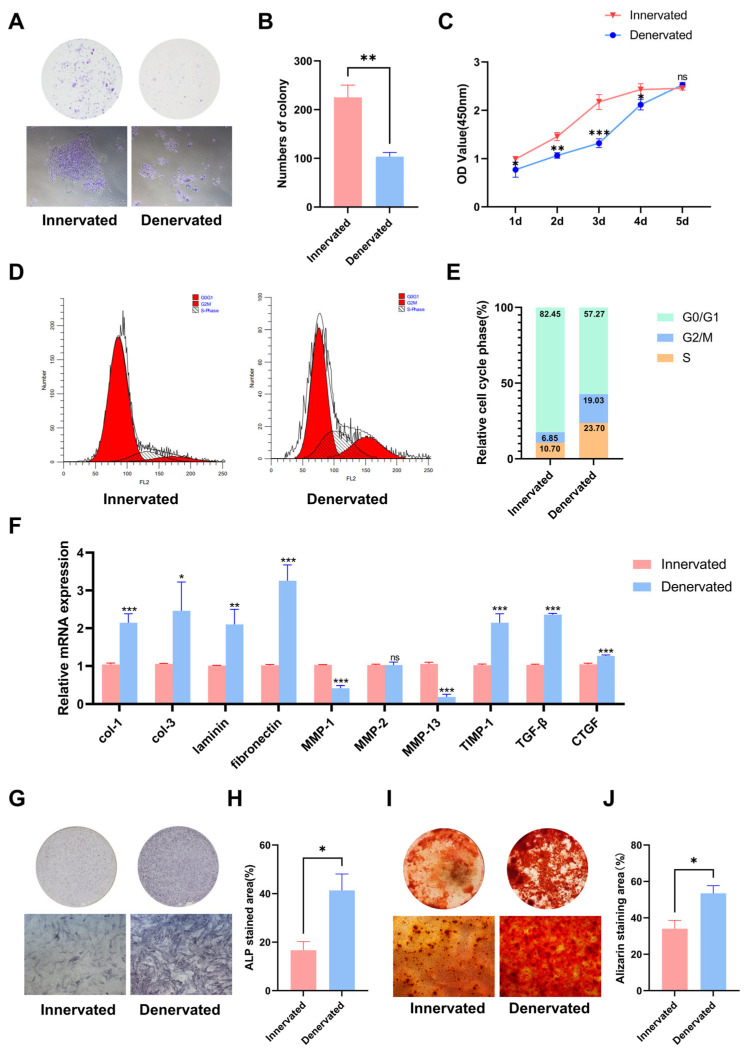
Denervated DPCs show cell cycle arrest and imbalance in synthesis and secretion. (**A**) Cell colony formation assay was used to analyze cell proliferation. (**B**) Quantification of the colony numbers in (**A**). n = 3 per group. (**C**) Cell proliferation was detected by CCK8 assay, and OD values are shown as the mean ± SEM (ns, no significant difference; * *p* < 0.05, ** *p* < 0.01, and *** *p* < 0.001; n = 3). (**D**) Flow cytometric analysis of cell cycle distribution in DPCs of innervated and denervated groups. (**E**) Distribution of DPCs among G0/G1-, S-, and G2/M-phases. (**F**) RT-qPCR analysis of mRNA col-1, col-3, laminin, fibronectin, MMP-1, MMP-13, TGF-β, and CTGF expression of DPCs. (**G**) Alkaline phosphatase (ALP) staining on day 7 after mineralization induction. (**H**) Quantification of the staining in (**G**) (* *p* < 0.05, n =3). (**I**) Alizarin red S (ARS) staining on day 21 after mineralization induction. (**J**) Quantification of the staining in (**J**) (* *p* < 0.05, n =3).

Because we observed that there was cell clustering in the dental pulp after denervation (Figure 1B), we examined the proliferation ability by using a colony formation assay and CCK8 analysis. The results showed decreased proliferation ability after sensory denervation (Figure 5A–C). To obtain further insight into the loss of proliferation ability, we examined the cell cycle status via flow cytometry. We observed that the cell cycle of the denervated DPCs was arrested at the G2/M phase, which has previously been shown to express cytokines to promote fibrosis (Figure 5D,E). 

Pulp fibrosis and pulp calcification arise from an imbalance in the synthesis and degradation of the extracellular matrix (ECM). DPCs play a crucial role in ECM synthesis and remodeling through the secretion of numerous proteins, proteinases, and cytokines [17]. To assess the ECM-related gene expression levels, we conducted an RT-qPCR analysis (Figure 5F). Our findings revealed a significant upregulation in the expression levels of the genes related to the extracellular matrix components, including col-1, col-3, laminin, and fibronectin. The mRNA levels of the matrix metalloproteinase (MMP) family genes MMP-1 and MMP-13 decreased, while the expression of the tissue inhibitor of metalloproteinase-1 (TIMP1) demonstrated an increase. Moreover, the expression levels of the cytokines, such as transforming growth factor-beta (TGF-β) and connective tissue growth factor (CTGF), which serve as crucial pro-fibrotic mediators, were elevated.

Additionally, we cultured the DPCs of both groups in a mineralization-inducing culture medium. Subsequently, alkaline phosphatase (ALP) staining was performed at 7 days, and mineralized nodules staining (Alizarin red) was conducted at 21 days (Figure 5G–J). The denervated DPCs exhibited elevated ALP activity and mineralization levels upon induction of mineralization. This suggests that the absence of sensory innervation renders DPCs more susceptible to mineralization under external stimulation, ultimately leading to calcification of the root canal.

## 3. Discussion

Pulp degeneration presents significant clinical challenges such as narrowed pulp cavities, massive calcified tissue in the root canal, diminished pulpal sensitivity, and reduced tooth resistance due to inadequate pulpal nutrient supply [28,29]. Therefore, investigating the mechanisms underlying the maintenance of pulp homeostasis is of utmost importance. Although previous studies have found the deceleration of incisor growth, the development of chalky plaque on the enamel surface, and the occurrence of calcification in the pulp chamber following the sensory denervation of rat incisors, it should be noted that rodent incisors possess a unique structural specialization that allows for continuous growth, posing challenges in replicating the pulpal conditions found in humans [30,31,32]. Thus, our study aimed to explore the role of sensory nerves in pulp homeostasis in rat molars, which bear a high resemblance to human dental pulp. We provide evidence that sensory denervation results in pulp fibrosis and calcification through the induction of DPC senescence and the disruption of ECM secretion and synthesis.

Sensory nerves constitute a crucial component of the dental pulp, and prior research has primarily examined their sensory function [33]. Recent investigations have revealed a notable decline in sensory nerve fiber density across various aging tissues, such as the skeletal muscle, cornea, and skin [34,35,36]. Sensory denervation may serve as a catalyst for tissue and cellular senescence, and it induces a histologic phenotype akin to that observed in aging bone and skeletal muscle [9,37]. Our investigations in dental pulp have yielded comparable findings, further supporting these conclusions. While sensory denervation induces notable pulp degeneration, the fibrosis and calcification patterns are not wholly identical to age-related pulp degeneration. In contrast to the primarily diminished chamber height observed in aged pulp, sensory denervation leads to reductions in both the height and width of the pulp chamber, accompanied by the emergence of irregular calcification growth centers within the root canal. This disparity may be attributed to the loss of innervated DPCs, which is progressive, hierarchical, and more regularly occurs with advancing age. Following inferior alveolar nerve transection, DPCs lose their sensory innervation simultaneously, resulting in cell senescence and functional imbalance everywhere in the pulp. 

Extended stimuli such as caries, attrition, abrasion, erosion, trauma, and surgical procedures establish a microenvironment that contributes to the development of pulp calcifications [38]. Our experiments showed that sensory denervation induces the senescence of DPCs, which are more susceptible to odontogenic/osteogenic differentiation in a mineralization-induced microenvironment, similar to chronological senescence. The mineralization capacity of DPCs increases in chronological senescence and decreases in replicative senescence [39]. Chronological senescence is an inherent and inescapable consequence of aging [39,40]. A significant distinction between replicative senescence and chronological senescence lies in the lack of an in vivo setting [41,42]. While an in vitro culture can supply the necessary nutritional support for cell culture, achieving innervation proves challenging; perhaps this is one of the reasons why replicative senescence is distinct from chronic chronological senescence. Additionally, telomere shortening is responsible for replicative senescence [43]. In contrast, the cellular senescence in this study is more similar to accelerated senescence due to extrinsic stimuli (premature senescence), which do not seem to be significantly related to telomere shortening [44]. Uncontrolled telomere lengthening is, by and large, at the expense of risking premature aging and related disorders [44,45]. In any case, our understanding of DPC senescence after sensory denervation needs to be further developed.

In this study, we utilized tissues and cells obtained two weeks after sensory denervation. The level of the senescence marker genes p53 and p21 was significantly increased following operation, while there was no notable change in p16 expression. p21 was upregulated during the early stage of senescence and p16 expression often appears later in the program, suggesting that sensory denervation may induce early cellular senescence [46,47]. Furthermore, our findings indicate that sensory denervation leads to cell cycle arrest in the G2/M phase. It is not inconsistent with previous reports [48]. The proportion of DPCs in G2/M phase arrest increases first and then decreases with age; DPCs of older adults arrest at the G0/G1 phase and have an inability to restart proliferation [49]. Sensory denervation is likely to accelerate senescence and may induce it to enter into the early stage of senescence. This is also in agreement with previous reports that p21 together with p53 causes cell growth arrest at the G2 phase [50]. 

Previous studies in renal fibrosis models have demonstrated that renal tubular epithelial cells arrested in the G2/M phase have an increased capacity to produce profibrotic growth factors, such as transforming growth factor-beta1 (TGF-β1) and connective tissue growth factor (CTGF), which aligns with the outcomes obtained in our current study of pulp [51]. This phenomenon can also be elucidated by the senescence-associated secretory phenotype (SASP), which encompasses various substances, such as collagen, laminin, fibronectin, matrix metalloproteinase, and cytokines [52]. However, our investigation into the expression levels of SASP in denervated DPCs does not entirely correspond with those observed in other aging cells, potentially due to variations in tissue specificity and cell specificity [53,54].

Pulp calcification is observed in the pulp of sensory-denervated and aging individuals, as opposed to osteoporosis-like changes occurring in sensory-denervated and aging bone. The calcification observed in the pulp may be comparable to the “calcification paradox”, which often refers to the paradoxical phenomenon of the simultaneous occurrence of vascular calcification and osteoporosis [55]. Research on the calcification paradox is currently limited, and there is insufficient evidence to support the notion that macrophages [56], various extracellular vesicles [55], caveolin-1 [57], vitamin K2 [58], and other factors play distinct roles in different anatomical sites. According to our research findings, which indicate the significant contribution of sensory nerves in maintaining pulp homeostasis and the distinct phenotypes observed in pulp with the bone following sensory denervation, it is plausible to propose that sensory nerves may have a role in the perplexing phenomenon of “calcification paradox”, either through direct or indirect involvement. However, further investigation is required to elucidate the precise mechanisms underlying this phenomenon.

Although the involvement of sensory nerves in the maintenance of pulp homeostasis was demonstrated at the histologic and cytologic levels, some limitations should be noted in the present study. However, the mechanism remains unclear and related protective factors should also be considered. Previous research has suggested that neuropeptides and neurotransmitters, such as calcitonin gene-related peptide (CGRP) [59] and substance P (SP) [60], play a significant role in the interaction between sensory nerves and their innervating tissues, but still, some studies have shown that sensory nerves also secrete other factors, such as sonic hedgehog (Shh) [61] and fibroblast growth factor 1 (FGF1) [32]. The identification of the specific substances responsible for maintaining pulp homeostasis is still uncertain. Furthermore, although DPCs have been selected as the primary focus of this study, it is essential to acknowledge their inherent heterogeneity, as different cell types possess distinct markers and functions [62]. Therefore, future research endeavors may identify subpopulations of DPCs that exhibit responsiveness to sensory nerves, aiming to enhance our comprehension of the underlying biological mechanisms within the dental pulp.

Interventions focusing on the aging process rather than specific diseases in individuals have the potential to prevent, delay, or mitigate the severity of various age-related diseases [63]. Our findings demonstrate that sensory denervation accelerates DPC senescence and pulp degeneration while ensuring that a sufficient innervation of the sensory nerves in the pulp may preserve the normal structure and function of the dental pulp. Presently, neurotrophic drugs are predominantly employed for the prevention and treatment of brain senescence and peripheral neurodegeneration [64,65]. Nevertheless, the potential viability of preserving the proper structure and functionality of the dental pulp through indirect neurotropism remains subject to ongoing investigation.

## 4. Materials and Methods

### 4.1. Experimental Animals

The Ethics Review Board of the Affiliated Stomatology Hospital of Tongji University (2022-DW-25) granted approval for all the animal experiments conducted. Male Sprague-Dawley rats, aged 8 weeks and weighing between 200 and 250 g, were obtained from SPF (Beijing) Biotechnology Co., Ltd. (Beijing, China). These rats were housed in a controlled environment with 12 h day/night cycles, maintained at an optimal temperature. They were provided with a standard laboratory diet and unrestricted access to drinking water. All the experimental procedures followed the ARRIVE guidelines 2.0. The animals were randomized into different groups, and at least 4 rats were used for each group unless otherwise stated.

### 4.2. Inferior Alveolar Nerve Transection

The sensory denervation procedure was performed based on previous protocols [30,66]. Under general anesthesia using 1% Pelltobarbitalum Natricum (P3761, Sigma-Aldrich, St. Louis, MO, USA), the masseter muscle was fully exposed through an extraoral horizontal incision. The mandibular bone surface was then revealed through careful dissection of the mas-seter muscle. A small dental round bur was utilized to remove the cortex bone and expose the inferior alveolar nerve, followed by the excision of a 5 mm segment of the nerve fibers. Care was taken to prevent damage to the blood vessels, and those who experienced unexpected bleeding were excluded from the study. The muscle and skin were subsequently closed and sutured. Each experimental group consisted of at least four animals. The rats were euthanized by intracardiac perfusion with 4% paraformaldehyde (PFA) buffered at pH 7.2–7.4.

### 4.3. Isolation and Culture of Rat DPCs

The primary DPCs were isolated following a published protocol [67,68]. The first molars were extracted from the mandible and subsequently cleaned of soft tissue using a scalpel. Dissection was performed at the interface between the root and crown to expose the pulp of the molar. The extracted pulp tissues were then subjected to digestion using collagenase type I (3 mg/mL, SCR103, Sigma-Aldrich, St. Louis, MO, USA) for 30 min at a temperature of 37 °C. Following digestion, the cells were evenly distributed onto 6-well plates containing α-MEM supplemented with 20% fetal bovine serum (FBS) and 1% penicillin-streptomycin. Cell passage 2 was used for the experiments.

### 4.4. Hematoxylin and Eosin (HE) Staining 

The experimental procedure followed the standard HE-staining protocol, which involved the initial fixation of the tissue samples in 4% paraformaldehyde (PFA) at 4 °C for 48 h, followed by demineralization in a 10% EDTA solution at 4 °C for approximately 2 months with a solution change every 3 days. Subsequently, the samples underwent dehydration through a graded series of ethanol, were embedded in paraffin, sectioned at a thickness of 4 μm, and stained using the HE-staining kit (E607318, Sangon Biotech, Shanghai, China). The resulting stained samples were then examined under light microscopy (Axiolab 5, Zeiss, Oberkochen, Germany). The pulp chamber size was quantified using ImageJ analysis software (Version 1.8.0, NIH, Bethesda, MD, USA). Briefly, the pulp chamber area (PCA) and tooth area (TA) were selected using the polygonal lasso tool by moving the cursor along their profiles. The ImageJ line tool estimated the pulp chamber height (PCH) and pulp chamber width (PCW). The landmarks used to calculate the PCH were the pulp chamber floor and the lowest part of the pulp chamber ceiling. The crown height (CH) was measured from the cusp’s highest point to the root bifurcation’s lowest point. The assessment of the PCW involved the utilization of a perpendicular line drawn through the central region of the PCH, spanning from the mesial to the distal walls of the pulp chamber. Similarly, the evaluation of the CW involved implementing a perpendicular line drawn through the central region of the CH, spanning from the mesial to distal walls of the dentin outside. The measurements were conducted on three separate occasions to ensure biological replication, and subsequently, the ratios PCA/TA, PCH/CH, and PCW/CW were calculated.

### 4.5. Scanning Electron Microscopy (SEM)

The pulp dentin internal morphology (the surface close to the pulp chamber) was characterized via an SEM (Supra 25-Zeiss, Zeiss, Oberkochen, Germany). The dentine samples were dehydrated via an ethanol gradient, fixed in 4% paraformaldehyde, then coated with a 20–30 nm thin metallic layer of gold in a sputter-coating machine and witnessed under an SEM. The accelerating voltage of the SEM was 20 kV, whereas different magnifications (based on convenience) were utilized.

### 4.6. Masson Staining

Masson’s staining was conducted using the trichrome Masson’s staining solution obtained from Servicebio (G1006). The myosin component was visually represented as red, while the fibrin component was depicted as blue. Subsequently, the stained samples were examined under a light microscope (Axiolab 5, Zeiss, Oberkochen, Germany).

### 4.7. Lipofuscin Staining

The tissues were subjected to fixation, decalcification, processing, and sectioning following the protocol mentioned above. Lipofuscin staining was conducted using the Lipofuscin Stain Kit (Long Ziehl–Neelsen Method) from Servicebio (G2020). Subsequently, the stained samples were examined under light microscopy (Axiolab 5, Zeiss, Oberkochen, Germany). For each group, the number of individual lipofuscin deposits were counted from 5 fields per slide.

### 4.8. Immunohistochemistry Staining

The tissues were subjected to fixation, decalcification, processing, and sectioning, as previously described [67]. An antigen retrieval was performed using hyaluronidase. The endogenous peroxidase activity was inhibited using a 3% H_2_O_2_ solution and goat serum. Subsequently, the tissues were incubated with the primary antibody at 4 °C overnight. After washing with PBS, biotinylated secondary antibodies were applied, and the samples were further processed using streptavidin peroxidase and a DAB Detection Kit (DAB-1031, MXB, Fuzhou, China) following the manufacturer’s instructions. The list of antibodies is shown in Table 1. Immunohistochemical quantification of the positive areas was performed using ImageJ analysis software (Version 1.8.0, NIH, Bethesda, MD, USA).

### 4.9. Immunofluorescence Staining

Immunofluorescence staining was conducted in cultured cells according to previously established protocols [67]. The cultured cells were fixed in a 4% paraformaldehyde solution for 15 min, followed by two washes with phosphate-buffered saline (PBS). Subsequently, the samples were subjected to blocking with goat serum at room temperature and incubated overnight at 4 °C with the designated primary antibody. The secondary antibody, conjugated with a fluorochrome, was then incubated for 60 min. The actin cytoskeleton was labeled with either phalloidin-AlexaFluor 488 (40736ES75, Yeasen, Shanghai, China) or rhodamine-conjugated phalloidin (40735ES75, Yeasen, Shanghai, China), and the nuclei were subsequently stained with DAPI (D9542, Sigma-Aldrich, St. Louis, MO, USA) for 5 min. The sections were affixed onto glass slides and subjected to microscopic examination. The list of antibodies is shown in Table 1. The percentage of cells with positive staining was determined using ImageJ software (Version 1.8.0, NIH, Bethesda, MD, USA).

### 4.10. Senescence-Associated β-Galactosidase Staining

The senescence-associated β-Galactosidase Staining Kit (C0602, Beyotime, Shanghai, China) was utilized to perform β-Galactosidase staining. DPCs were subjected to three washes with PBS and subsequently fixed for 15 min at room temperature. Following fixation, the DPCs were incubated overnight at 37 °C in darkness using the working solution. The DPCs were then observed using a bright-field microscope (Axio Vert.A1, Zeiss, Oberkochen, Germany). Blue-stained cells were considered positive for SA-β-Galactosidase; positive cells were counted and converted into a positive cell ratio.

### 4.11. RNA Isolation

Pulp tissues were ground in a liquid nitrogen-treated mortar, and the total RNA was extracted by Trizol (9109, Takara, Shiga, Japan) according to the manufacturer’s instructions. Cell lysates of both groups were harvested using Trizol. RNA was extracted by phenol–chloroform followed by isopropanol precipitation. The RNA concentration and purity were then measured using a NanoDrop ND8000 (NanoDrop Technologies, Wilmington, DE, USA). Samples with an OD260/280 reading between 1.8 and 2.0 were used. The total RNA was reverse-transcribed into cDNA using a cDNA Synthesis SuperMix for quantitative polymerase chain reaction (qPCR) with gDNA Eraser (11123ES60, Yeasen, Shanghai, China).

### 4.12. Real-Time Quantitative PCR

The real-time qPCR was performed according to the MIQE guidelines. We used a qPCR to quantify the mRNA levels. All the reactions were carried out in a total volume of 10 μL with 5 µL of SYBR Green Master Mix (Yeasen, China), 0.5 µL of each primer (10 µM), and 0.5 µL cDNA. The amplifications were performed in the LightCycler^®^ 96 device (Roche, Rotkreuz, Switzerland) using the following program: 5 min denaturing at 95 °C followed by 40 cycles of 10 s at 95 °C and 30 s at 60 °C, with a final stage of melting curve. The obtained data were normalized to the housekeeping gene β-actin, and the relative expression was determined using the 2^−ΔΔCt^ method. The primers were designed using Primer 6 software, and the primer sequences can be found in Table 2. All the experiments were carried out in triplicate.

### 4.13. Cell Proliferation Assay

The cell proliferation was quantified using a colony formation assay first. A population of 300 DPCs was initially placed in a culture dish, with the culture medium being replaced every 3 days. After 7 days, the resultant colonies were immobilized using PFA for 15 min and underwent crystal violet staining. After imaging the culture dish, the number of colonies was determined by utilizing the Colony Counter plugin on ImageJ (ImageJ, NIH, USA).

The cell proliferation was assessed by conducting CCK8 assays. The DPCs were distributed into 96-well plates, with each well containing 3000 cells. Subsequently, at 1–5 day post-seeding, the CCK8 solution was added to the wells and incubated for 1 h. The absorbance of proliferation at 450 nm was measured using a microplate reader (Bio-Tek, Hercules, CA, USA). All the aforementioned experiments were replicated three times.

### 4.14. Cell Cycle Analysis

The DPCs from both experimental groups were subjected to trypsinization in order to obtain a single-cell suspension for the subsequent cell cycle analysis. Following trypsinization, the cells were washed three times with cold PBS and re-suspended in 1 mL of PBS at a concentration of 10^6^ cells. Ethyl alcohol was subsequently added to the cell suspension while maintaining a low temperature. The cells were then cultured with RNAase for 30 minutes, after which one drop of propidium iodide (PI) solution (0.5 mg/mL, 1% Triton-100) was added before the flow cytometer analysis (BD Biosciences, San Jose, CA, USA). The resulting cell cycle data were calculated and analyzed using ModFit LT 4.0.

### 4.15. Alkaline Phosphatase (ALP) Activity Assay and Alizarin Red S Staining

The present study assessed the odontoblastic activities by utilizing alkaline phosphatase (ALP) and alizarin red. The rat DPSCs were cultured in 24-well plates and exposed to an odontoblastic differentiation medium, which consisted of 2 mM β-glycerophosphate (G9422, Sigma-Aldrich, St. Louis, MO, USA), 50 mg/mL ascorbic acid (A1300000, Sigma-Aldrich, USA), and 100 nM dexamethasone (D4902, Sigma-Aldrich, St. Louis, MO, USA). The medium was refreshed every 2 days, and after 7 days, the cells were fixed with 4% PFA and stained for ALP using an ALP-staining kit (P0321, Beyotime, Shanghai, China). Following a 21-day incubation period, the cells were treated with 4% PFA and subsequently stained with 0.2% alizarin red (A5533, Sigma-Aldrich, St. Louis, MO, USA) for 20 min at ambient temperature. The positive area was observed under a stereoscopic microscope (Axio Vert.A1, Zeiss, Oberkochen, Germany). The ALP- and alizarin red S-positive areas were quantified by ImageJ (Version 1.8.0, NIH, Bethesda, MD, USA).

### 4.16. Statistical Analysis

All the quantitative experiments were performed in triplicate. All the data are presented as mean ± SEM. (* *p* < 0.05; ** *p* < 0.01; *** *p* < 0.001; and **** *p* < 0.001). Student’s *t*-test was used for comparisons between 2 groups and the ANOVA test was to assess more than 2 groups. The analyses were performed with GraphPad Prism v9.0.0 (GraphPad Software, San Diego, CA, USA).

## Figures and Tables

**Table 1:** Antibodies used in this study.

Antibody	Species	Manufacturer	Catalog
Lamin B1	Rabbit	Boster	BA1228
COL-1	Rabbit	Boster	PB0981
COL-3	Rabbit	Affinity	AF5457
Laminin	Rabbit	Boster	PA1581
DSPP	Mouse	Absin	abs12771
α-tublin	Rabbit	Absin	abs619501
p53	Rabbit	Affinity	BF8013

**Table 2:** Primer sequences for quantitative reverse-transcription polymerase.

Target Gene	Primer Sequence (5′to3′Sequence)
p53-F	CTCCGACTATACCACTATCC
p53-R	GTCTTCCAGCGTGATGAT
p21-F	AGATGTGCCTATGGTCCTA
p21-R	TGTCTTGTCTTCGCTGAG
p16-F	CGATACAGGTGATGATGATG
p16-R	CTACCAGAGTGTCTAGGAAG
col-1-F	CGAGTATGGAAGCGAAGG
col-1-R	GCAGTGATAGGTGATGTTCT
col-3-F	CTACCTTGGTCAGTCCTATG
col-3-R	GCAGTCTAGTGGCTCATC
Laminin-F	AAGGTGTCTGGCTGGTTA
Laminin-R	GGTCGGTAGTGTCAATGTT
Fibronectin-F	GTAGCACAGAACTCAACCT
Fibronectin-R	CTCCTCCACAGCATAGATAG
MMP-1-F	GACCGACAACAGTGACAA
MMP-1-R	CATTAGTGCTCCTACATCTCT
MMP-2-F	ACAACCAACTACGATGATGA
MMP-2-R	TGGATAGTCGGAAGTTCTTG
MMP-13-F	GATGATGCTAACCAGACTATG
MMP-13-R	CTCTCACAATGCGATTACTC
TIMP1-F	TCTGGCATCCTCTTGTTG
TIMP1-R	GCTGGTATAAGGTGGTCTC
TGFb-F	CAACAACGCAATCTATGACA
TGFb-R	CAAGGTAACGCCAGGAAT
CTGF-F	TATGATGCGAGCCAACTG
CTGF-R	GCAGAAGGTATTGTCATTGG

## Data Availability

The data used and/or analyzed during the current study are contained within the manuscript or available from the corresponding author on reasonable request.
